# Integration of a Social Robot in a Pedagogical and Logopedic Intervention with Children: A Case Study

**DOI:** 10.3390/s20226483

**Published:** 2020-11-13

**Authors:** Verónica Egido-García, David Estévez, Ana Corrales-Paredes, María-José Terrón-López, Paloma-Julia Velasco-Quintana

**Affiliations:** 1 Vicedean Architecture, Engineering and Design Degree Programs, School of Architecture, Engineering and Design, Universidad Europea de Madrid, 28670 Villaviciosa de Odón, Spain; veronica.egido@universidadeuropea.es; 2 Aerospace and Industrial Engineering Department, School of Architecture, Engineering and Design, Universidad Europea de Madrid, 28670 Villaviciosa de Odón, Spain; david.estevez@universidadeuropea.es; 3 Science, Computation and Technology Department, School of Architecture, Engineering and Design, Universidad Europea de Madrid, 28670 Villaviciosa de Odón, Spain; anadelvalle.corrales@universidadeuropea.es; 4 Academic Model and Digital Transformation, School of Architecture, Engineering and Design, Universidad Europea de Madrid, 28670 Villaviciosa de Odón, Spain

**Keywords:** NAO robot, child–robot interaction, logopedic therapy, speech therapy, pedagogical therapy, adaptive behavior design

## Abstract

The effectiveness of social robots such as NAO in pedagogical therapies presents a challenge. There is abundant literature focused on therapies using robots with children with autism, but there is a gap to be filled in other educational different needs. This paper describes an experience of using a NAO as an assistant in a logopedic and pedagogical therapy with children with different needs. Even if the initial robot architecture is based on genericbehaviors, the loading and execution time for each specific requirement and the needs of each child in therapy, made it necessary to develop “Adaptive Behaviors”. These evolve into an adaptive architecture, appliedto the engineer–therapist–child interaction, requiring the engineer-programmer to be always present during the sessions. Benefits from the point of view of the therapist and the children and the acceptance of NAO in therapy are shown. A robot in speech-therapy sessions can play a positive role in several logopedic aspectsserving as a motivating factor for the children.Future works should be oriented in developing intelligent algorithms so as to eliminate the presence of the engineer-programmer in the sessions. Additional work proposals should consider deepening the psychological aspects of using humanoid robots in educational therapy.

## 1. Introduction

According to the World Report on Disability of the World Health Organization [1] 15% of the world population has some physical, mental, or sensory disability that makes it difficult to integrate themselves into the broader community. The report estimates that there are from 93 to 150 million children with disabilities in the world. Despite this statistic, there is little additional information about children with disabilities. The report recommends some action plans to remove barriers in order to improve their lives, such as integration projects in education. Artificial intelligence, in particular robotics, increasingly advances and proposes new solutions to make this integration easier.

Robotics is one of the fields with a greater variety of possible applications in all areas of science. With the appearance of more versatile robotic platforms and higher levels of programming, a clear path has been opened for the realization of pseudo-intelligent systems that help human beings in such different facets as industry, medicine, education, entertainment, disability assistance, construction, etc. In recent years, robotics has been expanding its field of action, from homes [2] to robots designed to facilitate and improve the quality of life of dependent people [3]. Within the latter, two distinct lines of work can be distinguished [4]: robots for rehabilitation (where there is no interaction between users and robots, and which are commonly used for physical disabilities); and social robots, which allow a human–robot interaction (HRI) in human society. The latter are those we use in this project.

Human–robot interaction (HRI) is a field of study that deals with the interplay of people and robots in a two-way communication in real-time. As this interaction requires communication, to have robots interact with humans is difficult unless a programmer is present [5]. Child–robot Interaction contributes in domains such as education, entertainment, and healthcare. This interaction with children sometimes requires a social dimension, in particular in education. Chita-Tegmark and Scheutz [6] consider socially assistive robots (SARS) those that aid users through social interactions rather than physical ones. However, despite the label, robots act socially only within limited scripted activities. Therefore, the use of social robots for education can be quite positive for learners [7] and how quickly the social robot responds is important. A robot that takes more than two seconds in giving an answer can induce the user’s frustration [8].

Social robots use social interfaces. They can be controlled remotely, and in some cases, they can be autonomous or have a human appearance. These characteristics facilitate their use in education, healthcare, and therapy [9,10,11,12,13]. They are programmed so that they can interpret certain social behaviors and perform other more automatic tasks such as playing sounds or gathering information. To do this, they must be able to interpret not only human speech, but also body language using computational psychology and advanced sensors to automatically recognize nonverbal cues. In addition, they must form part of the team with specialists not only in the area of robotics, but also in speech therapy and education.

The effectiveness of robots in educational contexts has been demonstrated by many researchers [13,14,15], as well as the benefits that the incorporation of robots with both human and nonhuman appearance can have in children with autism [16,17,18,19]. However, works referring to children with other educational needs are scarce [20,21,22]. There exists abundant literature focused on therapies using SARS with children with autism but there is a gap to be filled in logopedic and pedagogical therapies in other educational needs. Since each child is different, the robotic architecture must be flexible enough to fit a personalized therapy for the individual child. That makes it necessary to work closely with the therapists.

This paper looks on the integration of a social robot in a pedagogical and logopedic intervention with children. It is guided by the following questions:

- Are actual robots prepared to be incorporated into pedagogical and logopedic intervention with children?
- What are the requirements expected by the therapist?
- What hardware–software architecture best suits the requirements of those sessions?
- Will the initial results be encouraging enough for the continuation of the present project?

We present an experience of using a social robot in a logopedic therapy intervention in five children with different needs. The sessions were conducted individually. The therapist and the engineer-programmer were always present in the sessions with the robot and the child. We focus on the contribution of the robot’s appearance and behaviors.

This study aims to provide some guidance points for the research community on how social robots could meet the specific needs of logopedic and pedagogical therapies, as well as identifying points to consider to successfully implement them in this educational context.

The paper is structured as follows. In Section 2, our view on the use of robots in educational therapies with children through the lens of the literature is presented, explaining the robot and the platform that will be used. Section 3 describes the therapist’s requirements, the participants, and how the sessions took place. Section 4 explains how the initial architecture was modified into the final one in order to meet the requirements of both the children and the therapist. Section 5 gives the preliminary results based on the perceptions of the therapist. Finally, Section 6 presents some conclusions as well as suggestions for possible future works.

## 2. Background

The use of social robots in education has been found very useful in personalizing each child’s needs supporting individual learning across various domains [23,24]. Therefore, teachers and education therapists are considering humanoid robots as a useful tool for their practice [25,26,27]. This is because they are designed to be interactive with humans. Robots can be used to elicit cooperation in therapies in different ways:

- Following instructions, as in imitative games [28]. These skills play an important role in the development of social cognition and communication, not only in children but in human beings in general.
- Interacting with children. Children can increase their social skills using robots. For instance, robots can help children develop abilities such as waiting for a reply before talking. In addition, the interaction of children with robots from a nonverbal behavior perspective is possible too [29,30].
- Provoking unexpected situations. Breakdowns as not planned situations [31] as robots are unable to identify misunderstandings.

These actions must be programmed to be consistent with the needs of the therapies. Robots interact and communicate with children stimulating their learning. The robot can provide children the role of instrument and subject at the same time. Robots are task oriented and sometimes conversational. Hence, the robot has to understand children’s speech, which means that it requires a high-quality automatic speech recognition (ASR). In particular, in the case of children with disorders related to the language therapy field, robots offer an evolved capability for direct interaction with children [21]. Previous studies have shown that children with special needs tend to interact smoothly with robots as they are more predictable than human beings and do not express emotions, however, most of these studies were carried out with children with autism spectrum disorders (ASD). The use of humanoid robots such as NAO-type robots with children with attention deficit disorder (ADD), without or with hyperactivity (ADHD), and with speech language disorders such as dyslalias or dyslexias, is scarce [21,22,24,26]. In these cases, normally a support such as video and audio content are mostly used as a teaching media [32,33].

Since each child is different, the robotic architecture must fit a customized therapy for each particular case. Hence, robots are programmed to adapt their behavior to the needs of the children and the therapists.

The communicational interaction tools that NAO-type robots use for interaction, such as verbal skills or gestures, are less complicated than those of a human being. This is an advantage for therapies with children with learning difficulties. As NAO is programmed with predictable and simple conversational functions, it helps children to improve their communication skills.

### Robotic Platform

Aldebaran Robotics NAO (now SoftBank Robotics) was selected for the experiment as it is a robot that is widely used [34]. The NAO is a small bipedal humanoid robot with a friendly and nonthreatening appearance. What is more, it is well suited to work with children. It measures 50 cm height, weighs 4.3 kg, and has 25 degrees of freedom and comes with prehensile hands that can grasp and hold objects. It has a range of sensors and actuators: seven touch sensors, four directional microphones used to recognize sound from almost any angle, two cameras to identify the environment and recognize people, two loudspeakers, can speak through a text-to-speech engine (TTS), two cameras, a gyroscope, an accelerometer and a range of sensors (two IR and two sonars). It has an embedded computational core and connects externally via Wi-Fi or Ethernet. All these attributes allow for an attractive user interaction with the robot.

**Figure 1:**
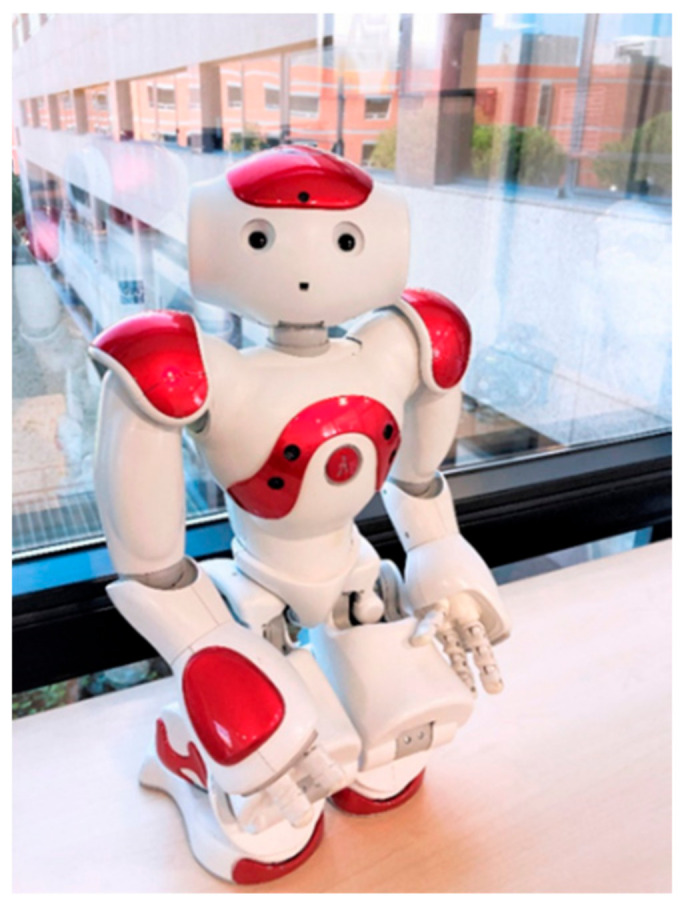
NAO robot ‘EBA’.

NAO has demonstrated to be a friendly robotic platform with children in several experiments such as [35], [36], and [37]. It is used with children in areas such as education [38], as well as emotional and behavioral analysis [39,40,41], physical therapy [27], health and social care [42,43], and autism therapies [44,45]. Based on its wide range of applications, we chose the NAO robot as a platform to provide help to therapists for the acquisition of language skills with the assumption that NAO is ready to be incorporated as an assistant into a pedagogical and logopedic intervention. We decided to give our NAO robot the name of EBA (Educational Behavior Aid). EBA (Figure 1) can provide support to the speech-therapist through educational exercises and recreational activities.

## 3. Method

For the research we followed a qualitative exploratory case study approach. Case study approach is adopted when we want to understand more about a phenomenon in context where there is little control over the events [46,47]. This happens when studying children’s behaviors. The purpose is not to make a comparison to other cases, just to explore the phenomenon in context. The interventions were implemented using the NAO robot ‘EBA’ in a therapeutic center.

### 3.1. Experimental Design

The team of researchers in charge of the experiment consisted of three robotic engineers, and three experts in education, one of whom has a degree in psychology. The robotics engineers are experts in robotic architecture and behavior programming systems, so they can make possible the requirements of the system. One of them oversaw programming the robot during the sessions with the therapist and the children (they will be called throughout the paper “the programmer” to differentiate them from the rest of the research team). Experts in education will be those who will guide the process and the robotic engineers in the development of the experiment and its design. They will serve as a bridge with the speech-therapist as a key piece for the continuous feedback and conclusions obtention of the experiment.

An initial meeting was held at the Therapist Center where on the one hand the objectives of the speech-therapist were established and, on the other, the researchers explained the potential benefits offered by EBA.

The speech-therapist, after analyzing these possibilities, considering all the agents involved in the process (parents and children) presented the intervention cases where the use of the robot could be helpful.

Before the study, precautions were taken to ensure ethics and protection of children. The study protocol was approved by the institutional ethical review board. The necessary procedures were made for the collection of the “Information sheet and informed consent” signed by the parents since the sessions with the minors were recorded. Parents were informed as to the purpose of the study and any questions they had were answered. The children’s verbal consent was also obtained prior to their participation.

### 3.2. Participants

A total of five children took part in our study. The children were chosen by the speech-therapist, after the meeting with the researchers, as being the one who knows how to work with each case. The cases were as follows:

Child 1 is 10 years old, with cleft palate, who has spent 5 years in speech therapy intervention. Child 1 has had various surgeries. Child 1 has problems in oral language, nasality, vocalization, and psychology in the use of language.

Child 2 is 9 years old, who has spent 3 years in speech therapy intervention. Specific language impairment (SLI), attention deficit hyperactivity disorder (ADHD) with comorbidity, dyslexia and oppositional defiant disorder (ODD).

Child 3 is 9 years old with SLI without attention deficit disorder (ADD), who has spent 3 years in speech therapy intervention. Child 3 has affectation at emotional level with misuse of oral language. Child 3 has had much involvement in semantic code.

Child 4 is 9 years old and has spent 3 years in speech therapy intervention. Child 4 has a language developmental delay and several types of dyslalia.

Child 5 is 12 years old and has spent 3 years in speech therapy intervention. Child 5 is dyslexic with ADD.

### 3.3. Environmental Setup

**Figure 2:**
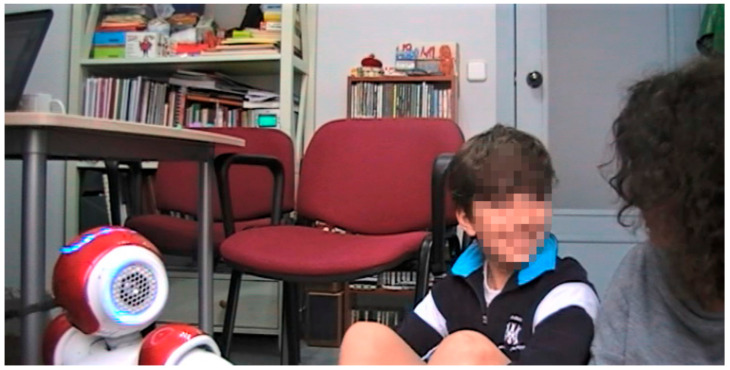
Experimental setup during a therapy session with ‘EBA’.

Interaction sessions always took place during ordinary therapy sessions at the speech-therapist center. They were conducted in a 5 × 4 m room (Figure 2). At each session, the therapist was always present at each session as the children interacted with ‘EBA’ as well as the engineer member of the research team in charge of programming ‘EBA’. Therefore, we refer to this member of the research team as the programmer. The researcher-programmer always sat in a corner on one side of the table, with ‘EBA’ nearby looking at the child. The therapist and the child were in front of him and could move around the room. A small video camera was placed behind the programmer to record the sessions. The camera was arranged so that it was almost imperceptible to children so that it did not influence their behavior.

Before the session the programmer was in the room to make sure that everything was working properly; in order to try to prevent disruptions during the session. Afterwards, the programmer recorded in his personal diary what he experienced during the session for later analysis and review.

The child interacted directly with the robot and with the therapist. The speech-therapist-educator established the intervention objectives in the different case studies, depending on each subject.

After each session, the programmer and the therapist recorded in their personal diary about what they experienced. These diaries were very different as the programmer one was more focused on technical objectives and the therapist one on pedagogical ones.

Based on what we learnt in each session we structured the next one to achieve the therapeutic objectives.

### 3.4. Data Collection and Analysis

The experiment was conducted once a week for 30 weeks. Each session started by greeting the child and lasted about 30 min. Over all the sessions with the five children we recorded about 180 min with EBA. Due to personal reasons some children could not attend all the sessions.

To gain in-depth understanding about each case and about the process followed during the therapy sessions, two semi-structured interviews were conducted to the speech-therapist: one before starting the experiment and one when the experiment was finished. The objective of the first interview was to achieve a better understanding of the sessions and the interventions with the children to adjust the robot programming. The second one helped us to review the progress of each child with respect to the beginning and fix some aspects (categories) related to the behaviors of the children with the robot that had been observed in the video recordings and in the reflective diaries, in order to get a better understanding of the context.

The interviews were conducted by the researcher who, in addition to being a psychologist, is a specialist in educational research. Semi-structured interviews allow to ensure that all the topics are covered allowing flexibility to the interviewee. The interviews were recorded.

Therefore, multiple sources of data were triangulated to give validity to the case study. The data gathered for the experiment were:

- Therapist personal diary of each session;
- Programmer personal diary of each session;
- Semi-structured interviews conducted to the therapist;
- Video recording of the sessions.

Diaries, interviews, and videos were qualitatively analyzed through coding and interpretative analysis techniques using NVivo13. They were analyzed as follows:

- A verbatim transcript of the content of the interviews and videotape recording.
- Data analysis with a reading of the transcribed material and viewing videos, in order to identify content units and establish initial categories.
- Successive meetings to establish the dynamics of coding, review the material, and resolve by consensus the observational categories. Discrepancies were resolved by discussion.
- We agreed during the process a “book of categories” in which a brief description of the specified category, an expanded definition, criteria about when using the category, and its grouping in families. This book allowed a more global view of the categories (parent nodes) and emerged families (tree nodes) of the analysis.
- This process was completed by saturating the information.
- Finding important quotes that describe the main ideas, grouping them to examine the general ideas, and finding the correlations between them.
- Constructing a narrative that connects the findings.

## 4. Architecture: Description and Evolution

The ‘EBA’ architecture pursues meeting the therapist’s requirements while reaching a rich child–robot interaction.

To program the robot’s behaviors, we used Choreographe, which is a visual programming tool based in C++ and Python. It allows to program animations and behaviors in ‘EBA’ and tests them on a simulated environment before trying them with real users.

A behavior is a set of instructions that can be coded in the robot. In this work, the design of each behavior was carried out once the therapist determined the specific needs in each of the five cases of study.

### 4.1. Initial Architecture

**Figure 3:**
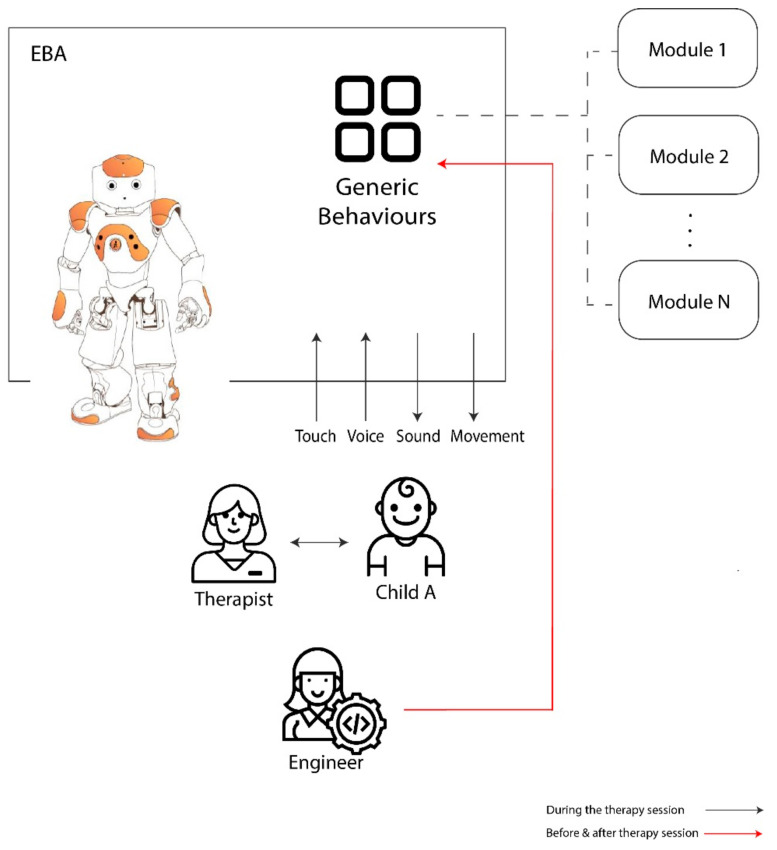
Initial architecture of the model applied to the engineer–therapist–child interaction.

Knowing the requirements of the participants, we agreed to start with a common initial architecture for all cases. The initial architecture of the model applied to the engineer–therapist–child interaction is presented in Figure 3.

During the session, the child interacts with EBA robot (human–robot interaction HRI) and the speech-therapist (human–human interaction HHI). The inputs for the HRI system are tactile sensors and voice, while the outputs are sounds and movement.

Generic behaviors are all those that are common in all cases. To define them, it is necessary to hold meetings between the therapist and the engineers, with the aim of analyzing the characteristics of each child and to specify common needs before the children interact with the robot. As a result, EBA was preprogrammed with the following generic modules:

- Reading comprehension;
- dictations, stories and vocabulary, improvement of oral comprehension;
- articulation and phonetic-phonological pronunciation;
- phonological awareness and phonetic segmentation;
- literacy skills.

The content of each module was decided upon discussion with the speech-therapist based on her experience in previous sessions with children. The objectives pursued in each module of the sessions and the EBA mission in each one is presented in Table 1.

As the therapist and the programmer had planned beforehand, during the first sessions, the different modules were activated. They were scripted as theatre for the children as the instructions and their sequences were preprogramed.

**Figure 4:**
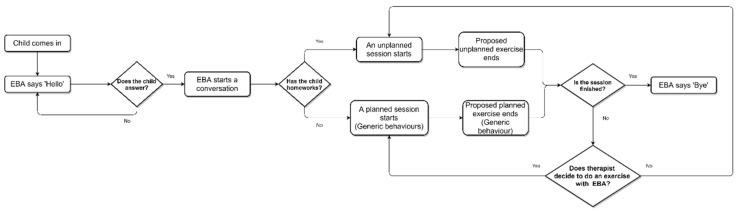
Sequence of a session with the initial architecture.

In Figure 4, the sequence of a session, related to the initial robot software architecture, is shown. The robot starts the interaction. ‘EBA’ asks the child social questions such as: “how are you?” or “how is school going?”. If the child has homework, the therapist will start the unplanned session, the robot will not be part of the proposed exercise. If the child does not have homework, the therapist will proceed to do planned exercises with the robot, that is, an exercise (or exercises) using the generic behaviors of the robot will be proposed. The NAO executes the generic behavior modules, depending on the therapist’s guideline. If the session has not finished, the therapist will decide whether to do an exercise with the robot, to reinforce the work done during the session (planned session), or to continue with the unplanned session.

The difficulty of keeping the pre-established sequence was detected due to the unpredictable behavior of the children, the sluggishness of the software (a faster response by the robot was needed), and the change in the behaviors that EBA causes in the children (different in each child). After each session, the engineer received feedback from the therapist about how the objectives of the session were met and possible changes that could be made according to the process lived.

The initial conclusions were that therapy should be adapted in real time in each intervention as children showed different needs each time. These conclusions implied changes in the software architecture of EBA and the modules that would have to be faster and adapted to unpredictable scenarios. Therefore, a software modification was made, prioritizing lived experience and the objectives that would be sought in the following sessions. In the next section, we will explain the change in our system architecture to an architecture that includes adaptive behaviors.

### 4.2. Final Adaptive Architecture Description

The observation in the first sessions with children was that the static architecture initially proposed did not achieve the requirements established in the therapy. Children showed a variability in their behaviors, only a real time adaptive system would allow children to keep their attention on the EBA robot and achieve the therapeutic goals prescribed for each session.

**Figure 5:**
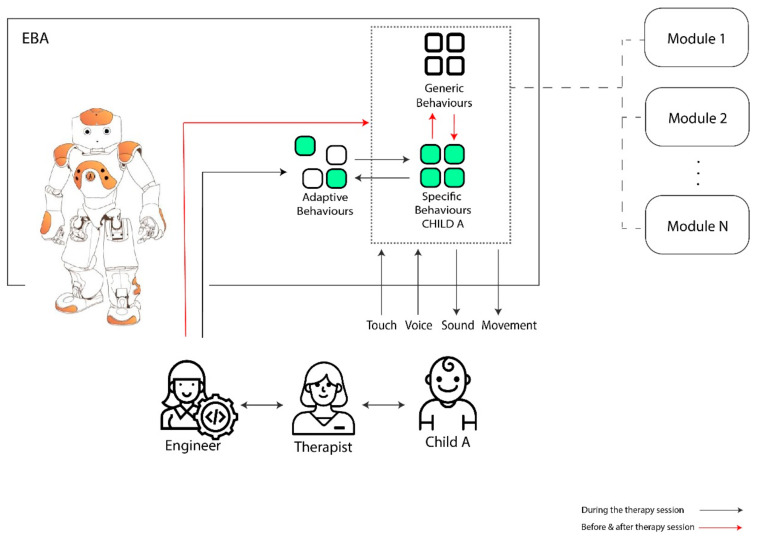
Final adaptive architecture.

The new architecture of the model applied to the engineer–therapist–child interaction is described in Figure 5. This architecture is based on an adaptive system oriented to satisfy the changing needs of the children and the speech-therapist throughout the intervention. This architecture is based on a modular system designed to benefit the robot–child interaction [5].

As in the previous model, during the session the child interacts with the EBA robot through tactile sensors and voice, with outputs being sounds and movement. The therapist interacts with the child as well. The question remains, how do the new modules of “Adaptive Behaviors” and “Specific Behaviors” improve the development of the session?

Attending to the needs of the child, the engineer becomes an active participant during the session. In order to attend to the needs of the children, the engineer makes adjustments in EBA in real time. Faster responses were given with the orientation of the therapist and the previous experience with the child. The “Generic Behaviors” are maintained as the central part of the therapy but the system adapts itself with changes. The unpredictability reactions of each child in therapy, made it necessary to include the “Adaptive Behaviors” in the robot. Sometimes the adaptive behaviors are activated randomly to see what the reaction of the child might be. Evaluating the child’s reaction, the alternatives considered include discarding that behavior or converting it into a preprogramed one.

As an adaptive behavior becomes a useful module for the child’s session it ends up becoming a specific behavior for that child. The “Specific behaviors” block contains modules adapted to the distinctive features of each child. Some of the “Generic behaviors” ones could change to “Specific behaviors” if they are not valid for all the cases. Inversely some of the “Specific behaviors” could change to “Generic behaviors” if these are valid for all the children.

The “Adaptive Behaviors” allow for the interaction of the Engineer in the session, permitting the modification of the specific modules in real time. Thus, the flexibility of the architecture constantly increases as a whole. For example, if, during a session, the therapist must modify the planned therapy guidelines, due to an unexpected response from the child, the engineer can modify the robot’s behaviors in real time. Introducing the input manually improves the velocity of the answer. Further, during the session the therapist can request to have a reaction in EBA included in some not preprogrammed inputs and the engineer should comply promptly.

Adaptive Behaviors allow the therapist to test and evaluate in real time the child’s reactions to new robot actions. The therapist can then decide if these Adaptive Behaviors can become Specific Behaviors for a particular child’s therapy.

#### Example of an Adaptive Behavior and/or Specific Behavior

During the interaction with the child, EBA can speak and express a series of phrases adapted to each case. A long list of possible utterances was compiled in a different database which the robot can access. Each utterance was assigned to a particular category such as ‘greeting’, ‘question’, ‘feedback’, etc. As EBA was limited in its expressiveness some additional behaviors were implemented. These included for instance some gestures and sounds such as raising the arms or conveying different forms of feedback to reflect happiness or excitement.

The improvements of the different generated modules were due to the feedback from the children and to the observations made in the sessions. These included encouragement or motivational sentences for the children to persuade them to continue working. Going deeper into the subject matter was inevitable as we worked continuously for the gradual improvement of children involved in the project. More complete or elaborated histories are implemented to observe the improvement of the attention of the children when they work together with the EBA robot.

## 5. Preliminary Qualitative Results and Discussion

To explore the interaction of children with EBA, a qualitative study was done. In this section we explore some results, from the qualitative analysis of this study, focused on adaptations made in architecture in order to meet the requirements of the sessions. However, specific competences development related to the children logopedic and pedagogical progress are out of the scope of this paper. Results come from the constructed narrative that connects the findings from the qualitative analysis of this study. We divided these into two sections: technical observations and our learning from the sessions with the robot.

### 5.1. Technical Observations

One of the main results obtained in this study was the need to develop adaptive behaviors that met the needs of the sessions.

Adaptive programming is given by the needs of the user. In this case, the needs are those of the actors in the study: the children and the therapist. However, it is not possible to work with generic behaviors in the robot for all cases. Each child has specific needs. An action may motivate a child but may have the opposite effect in another child. The analysis of the therapist’s script and the experiments led us to modify our initial software architecture and include ‘Adaptive Behaviors’. These behaviors were tested with the child in a session. If the child’s feedback was positive for therapy, in consensus with the therapist, these behaviors went from being adaptive to become specific behaviors during this specific child’s session. These changes are motivated by different aspects detailed below.

The engineer and the therapist realized that to have a unique software for all the children is complicated. Each one has individual issues to deal with and different ways of approaching the different needs. Therefore, if there is only one software for all the cases, it would be very heavy when loaded. Each case implies a different approach inside the software. This slows the response of ‘EBA’ and therefore the child may lose the communication or connection he had with the robot, and therefore the interest generated in that session. As a result, we concluded that we must create Adaptive Behaviors for each child.

Something the therapist told us that we also had to consider was that the child responses were diverse in every session since their mood and vital time is variable. Therefore, you cannot always follow a preset script. Again, this requires a quick reprogramming of the robot time to suit the needs of the child during the session.

Besides, having a single software means that the robot stores variables that generate sentences that could be disruptive. As a result, sometimes children do not understand ‘EBA’ or do not understand why ‘EBA’ has come to that sentence.

The robot sometimes does not hear the order (due to ambient sound or pronunciation of the child) and the engineer must introduce it manually so that the response is quicker and accurate. Hence, it seems that there is “real” conversation between child and robot which is fundamental for simulating an adaptive social interaction between them.

The spontaneity faced by NAO in the sessions makes it not possible to program the robot beforehand. If suddenly, EBA is asked questions that are not included in the initial program, it must be reprogrammed quickly. Sometimes, this response time can be up to two minutes which can lead to frustration in children. Hence, the child can lose interest or fail to focus attention on EBA. Therefore, we must look for a way to reconnect with the child again. To improve this responsiveness range and speed of programming, it was decided to go further dividing the different programs used into adaptive behaviors. The very heavy burden of software based on EBA is the greatest impediment to rapid response.

With this, various more specific behaviors such as math, language, stories with questions–answers, and others more focused on social dialogue were developed. In addition, the specific topics discussed in the sessions were agreed with the speech-therapist in order to have a preprogrammed part. In that way, if new themes appear during the sessions, this allows the engineer enough time to make the needed changes.

A new way of programming based on agile software theories starts. Thus, the software is separated into several cases:

- individualized software per children;
- individualized software per disorder;
- individualized software per type of work to be done in the session.

With this new way of programming sessions, loading the software in EBA is faster. Furthermore, when the session is not following the initial approach due to external reasons (child with school problem, exams, arguing with teachers, etc.), the software can be modified faster. Thus, ‘EBA’ achieved a better adaptation to the environment, creating in the child “an illusion of better understanding”.

Besides, by creating different algorithms for each case, feedback is given to the software. Thus, the response is faster, and knowledge of topics of interest can be extended. The therapist commented on the importance of this as the fact that the robot adapts its behavior to each case (one software per child). Response times are reduced, and their social interaction is improved by programming a series of responses that strengthen the confidence depending on the child.

With the therapist center, just as it is agreed, we tried to follow a script session whenever was possible, trying to avoid such adaptability (and frustration) that can lead the child to lose interest in EBA for its belated response to the problem proposed.

Throughout the process, the sessions had to adapt to each case. A limitation observed in the sessions was that at times it was required a greater capacity of movements in the robot, but ‘EBA’ must be always connected. EBA mobility is reduced to a controlled area near the programmer.

The NAO robot has movement, but does not express feelings, therefore, the nonverbal communication of EBA is complicated. Therefore, we valued to endow that expression with colors in the eyes, as it is a feature of NAO. However, during the sessions, the therapist told us that this deficiency is detected as something positive for our case studies. This absence of nonverbal communication caused children to move and act more freely because they did not feel judged or valued by EBA. EBA just gives oral feedback with short phrases such as “Very good”; “Well done”; “Keep it up” and they preferred this feedback to the one given by the therapist. The incorporation of a robot as EBA in the context of intervention speech therapy for children with difficulties in oral language shows some elements of great value as work with structured language and the absence of value judgments, such as an objective response of EBA. As an element to deepen the emotional absence of EBA appears and how this absence impacts on the relationship with the children.

### 5.2. Learnings from the Sessions with the Robot

To explore the interaction of children with EBA a qualitative analysis was done using the gathered data from:

- Two interviews with the speech-therapist about the process (one before starting the experiment and one when the experiment was finished);
- Programmer and therapist diaries about the sessions;
- Video recordings of some sessions of children with EBA.

Some categories emerged from those sources through coding and interpretative analysis techniques using NVivo13. In this section we will only refer to those related to the interaction of the children with the robot ‘EBA’. The categories are bold highlighted in the following paragraphs. We included some quotes (in italic) from the second interview with the therapist about these categories.

It seems that incorporating a robot in the speech-therapy session can work positively as regarding **the formulation of questions and answers:** it implies developing the ability to understand and to build sentences. On the one hand, the child should strive to pronounce and organize the information in a consistent, clear, and simple way so that the robot understands the question. On the other, only answering properly, the child receives a positive feedback from EBA. As a result, it is perceived as an improvement in **understanding long sentences** which is attributed by the speech-therapist to the use of the robot (EBA) as a support tool.

Additionally, in cases where the objective is that the child increases his **volume of voice**, EBA achieved positive results; the child struggles to be heard by EBA and naturally (and unforced) gradually increases the volume of the voice.

*‘... It was a moment that ‘EBA’ said “Speaks me higher” and the child saw that EBA didn’t respond because he could not hear. She started up inadvertently, i.e., not gave the command “turn up the volume of your voice” because she has fully retracted that order. But she needed EBA listening to her, then there appeared the volume. So, there has been a significant change and from there it is true that parents have spoken with me and they say they have noticed that she speaks a little higher....’* [Second Interview with speech-therapist]

The therapist also considered that the incorporation of EBA at the session is a **motivating element** for the children. They have a greater willingness to learn and try to get it, and therefore they strive to have higher performance. Regarding “to follow instructions”, the speech-therapist notes that several children are more willing to learn (and work, to perform the exercises) when EBA is present.

“*She tells it to her friends in class and had to send a photo of the robot…. She told me that she is one of the few children in the school who has a robot. She says so and therefore she does her best to work or play with EBA.*” [Second Interview with speech-therapist]

As a result, the therapist notices improvements regarding **attention**, both sustained (that is maintained for a certain time period), as targeted. This makes distractions removed from the session, which undoubtedly positively influences the use of it, and therefore performance.

Elements not initially considered as a target appeared and can be significant for future work proposals. There are **psychological aspects** that could be incorporated in a context of a multidisciplinary intervention. Sometimes children show some concern for the welfare of the robot or give an emotional response: they start **creating a link with EBA.** This aspect would need further study and development, in particular, aspects of nonverbal communication and emotions such as empathy.

“*I remember one day in a session were EBA had a broken finger, … Well, the parents had to phone me! Their child was scared because we had put an electrical tape on EBA finger, and he thought it was a cast. He had normalized EBA as if it were human. When the parents phone me they told that their child had slept badly, that “poor EBA”,…, that “Daddy please, phone EBA to see how is she, because she is in the hospital for sure….” But of course, you have toget to know this child. That this child says that and sees a fellow man as his fellow man..., because his equals... well, he has many behavior problems because he has an oppositional defiant disorder. So, normally, he attacks his peers. His greatest communication is to attack…. And suddenly, being so worried about EBA? It was a great leap that has also taken him out. That is very nice, it moves me.*” [Second Interview with speech-therapist]

**Behavioral changes** are observed in the relationship that the children establish with EBA throughout the process. Curiosity, but also suspicion and even fear, appear while the sessions progress. This is reinforced by the possibility for children to have different relational roles with EBA: teacher, student or equal.

“*He likes to show that he is smarter than the robot*” [Diary of the Therapist].

In some cases, children placed EBA as a resource, sometimes also with ambivalence (sometimes showing empathy and other ignoring EBA). As for **interaction styles**, EBA allows not only that children have a figure more to learn from (another source of resources/content). The use of the robot allows children to take the role of teacher and be who teaches and transmits content. Diversity was seen in the relationship established with EBA, depending on the age and maturity of the child. In some cases, they see the robot as a tool, while in other cases, it is seen as an equal (personifying), etc.

“*For Child 5 EBA looks like a robot. Child 5 is older than the others (12 years old). It is the only case that sees EBA as a robot. As a resource to learn. That is, child 5 is not going to become a friend of EBA.*” [Second Interview with speech-therapist]

An item not initially considered, but that seems important, is the **relationship** not only **with the speech-therapist** (which was expected since it is the reference person for the child, known and who maintains a significant degree of confidence), but **with the programmer**. This link has helped in the integration of EBA in the sessions and in the work of achieving the objectives listed.

Regarding the interaction **with the programmer**, most of the children ignored him (David), as if he were not there:

“*Well, David is part of the furniture in the sessions. In mean, the children just greeted me and EBA. At the beginning I had to tell them, ‘Hey this is David, greets him’ … “ but then I gave up, because I realized that, for the children, he didn’t exist directly. Look! Not only did they not be aware that David was there, but they didn’t even realize there was a camera recording them*” [Second Interview with the speech-therapist]

However, sometimes, the children asked him questions about the robot, such as: *Did it sleep well? Does it have siblings? or Why don’t you build its parents?* However, always related to the robot. It seems that for the children the programmer is someone needed for the presence of the robot there.

## 6. Conclusions and Future Works

In this work, our main objective was the integration of a social robot in a pedagogical and logopedic intervention with children. Throughout this study we observed the advantages and limitations of incorporating a NAO type robot, ‘EBA’, as an assistant for the speech-therapist. After several trials, we managed to incorporate the robot into the therapeutic process. We learned from each experiment and each session.

From the point of view of the therapist, ‘EBA’ has fulfilled the requirements needed for the development of the pedagogical and logopedic sessions. According to the therapist’s assessment, the use of a robot as an assistant in the therapies’ sessions bring about a positive response from the children because it motivates them. We came to see that a robot can play a supporting role in the sessions having a different effect than the therapist one. All these facts have made the robot a good learning tool for children and it has been a useful tool for the therapist to improve the observations of each session and discover new elements to include in each child’s therapy.

Based on the analysis of qualitative data, from the point of view of the speech-therapist, the strengths of the EBA robot in relation to the improvements perceived in the worked sessions with the children are:

- Structured language: EBA’s language is structured in a better way. The speech-therapist attributes certain positive effects on children to this.
- Absence of value judgments: According to the speech-therapist, thanks to the objective responses of EBA, the value of the reinforcer emitted by EBA to the children is higher.
- While the children see ‘EBA’ in the form of an equal (expressing some kind of empathy towards it), or as its student (the child becomes the teacher), some changes can be done in the way of working the objectives of the sessions. This turns into an amelioration of the children vocalization, improvement in construction and structures of sentences and, as a result, in an increase of the self-confidence of the children.
- The use of a robot in the therapy is helpful as a good learning tool for children and as a useful tool for the therapist to improve the observations of each session to discover new elements to include in each child’s therapy.

The therapist contemplated meaningful application possibilities; some limitations were detected.

To get an idea of usability and feasibility aspects, the number of participants provides worthwhile insights. Perhaps, future studies will compare similar sessions in other similar therapy centers in order to study if the use of these robots has better results when we focus on specific needs.

As ‘EBA’ should always be connected, this gives the robot a restriction of movements. This was a limitation in those cases in which the children expected more movement in ‘EBA’, decreasing their motivation in these sessions.

Regarding the execution of the robot’s behaviors, to launch the software takes a long time when just using the Generic Behaviors, previously defined with the therapist. These behaviors included actions valid for all the cases. This time was shortened by implementing Adaptive Behaviors into the robot, because each child had specific needs. In this way, the system allows in situ changes during therapy, in a clear process of adaptation. These changes made during therapy, with precise instructions from the therapist, once tested, could become Specific Behaviors for the case under study.

Additionally, it must be considered that, in therapy sessions with the robot, the figure of the engineer-programmer must always be present. NAO is a robot that does not develop new strategies on its own, but rather its decisions come from what was previously programmed. The presence of the programmer during the session is necessary to adapt the architecture in real time to fit the child and the therapist requirements. It must be considered that at the beginning of a therapy, it is not known how the children are going to behave. The course of the sessions with EBA depends on the mood of the children at specific moment. Consequently, at least during the first sessions, collaboration of experts from both areas of knowledge seems to be required.

Once the use of NAO has been decided and a flexible software architecture has been designed, the next steps should be done in order to create a more autonomous system. Not having a programmer in each session should be explored as future work in two different fields: one of the fields related to the robot and the other related to the role of the therapist. Related to the robot, the hardware and software characteristics should be improved. For example, a better processor and more memory, better sensors in speech recognition, and visual social signal processing are needed.

Advances in machine learning technologies will enhance this field of study. Progress should be directed in this field focusing mainly on allowing the robot to create its own behaviors (learning of each session). An adaptation of the robot to the different sessions with different children, and related to the role of the therapist, creating a user-friendly interface for the therapist should be done. The therapist should be able to control the robot during the session autonomously, without the presence of the programmer, in order to give the key to get the needed autonomy of the system.

This software interface, oriented to the control of the robot by the therapists, can be the key to achieve the incorporation of robots to therapies. Once this objective is achieved, the programmer would become a software updater, and his role would be less binding.

Findings suggest that the use of social humanoid robots in logopedic therapies is promising and it opens many possibilities to continue working on introducing robots to improve the interventions in the speech-therapy centers. With the necessary adjustments, additional research examining the use of NAO by speech-therapists in other centers and with children with other profiles should be done. Understanding the perceived benefits and challenges of this case study can inform other speech therapy centers how to perform similar experiences.

## Figures and Tables

**Table 1:** Modules with the pursued objectives and the mission of EBA in the module.

Module	Objectives	EBA Mission
Reading comprehension	To strengthen reading	To ask questions about the text that has been read
	To work memory and sustained attention	To give positive feedback to the child
Dictations, stories, and vocabulary. Improvement of oral comprehension	To strengthen vocabulary, as well as written and oral comprehension	To tell a story
		To ask questions about the story
		To dictate To check spelling To give positive feedback to the child
Articulation and phonetic-phonological pronunciation	To develop the grapheme-phoneme correspondence.	To assume the role of the student, the child assuming the role of the teacher.
	To recognize and to identify the auditory discrimination of the different phonemes.	To indicate “understand” or “not understand” to the child to give feedback.
	To develop pronunciation skills.	To assume the role of the student, the child assuming the role of the teacher.
Phonological awareness and phonetic segmentation	To strength the lexical and phonological routes of reading and writing.	To say letters to get the child to say words starting with those letters.
	To enhance the grapheme-phoneme conversion.	To say words for the child to say how many syllables each word contains.
	To identify syllabic structures cv, vc, cvvc, cvcv, ccv, cvv, ccvc.	To give positive feedback to the child.
Literacy skills	To increase the concentration of the child and strengthen auditory processing.	To ask the child to repeat more clearly what he/she has said.
	To be able to respond to a verbal question.	To ask questions about a listened story.
	To develop active listening skills.	To give instructions to the child for all the activities defined.

## References

[1] (2011). WHO World Report on Disability. http://www.who.int/disabilities/world_report/2011/en/.

[2] Gates B. (2008). A Robot in Every Home. Sci. Am..

[3] Van der Loos M.H., Reinkensmeyer D.J., Guglielmelli E., Siciliano B., Khatib O. (2016). Rehabilitation and Health Care Robotics. Springer Handbook of Robotics.

[4] Broekens J., Heerink M., Rosendal H. (2009). Assistive social robots in elderly care: A review. Gerontechnology.

[5] Belpaeme T., Baxter P., Read R., Wood R., Cuay’ahuitl H., Kiefer B., Racioppa S., Kruijff-Korbayov’a I., Athanasopoulos G., Enescu V. (2012). Multimodal Child-Robot Interaction: Building Social Bonds. J. Hum. Robot. Interact..

[6] Chita-Tegmark M., Scheutz M. (2020). Assistive Robots for the Social Management of Health: A Framework for Robot Design and Human–Robot Interaction Research. Int. J. Soc. Robot..

[7] Belpaeme T., Kennedy J., Ramachandran A., Scassellati B. (2018). Social robots for education: A review. Sci. Robot..

[8] Shiwa T., Kanda T., Imai M., Ishiguro H., Hagita N. How Quickly Should Communication Robots Respond? In Proceedings of the Third International Conference on Human Robot Interation—HRI′08.

[9] Abbas T., Khan V.-J., Gadiraju U., Barakova E., Markopoulos P. (2020). Crowd of Oz: A Crowd-Powered Social Robotics System for Stress Management. Sensors.

[10] Song Y., Luximon Y. (2020). Trust in AI Agent: A Systematic Review of Facial Anthropomorphic Trustworthiness for Social Robot Design. Sensors.

[11] Martín A., Pulido J.C., González J.C., García-Olaya Á., Suárez C. (2020). A Framework for User Adaptation and Profiling for Social Robotics in Rehabilitation. Sensors.

[12] Dawe J., Sutherland C., Barco A., Broadbent E. (2018). Can social robots help children in healthcare contexts? A scoping review. BMJ Paediatr. Open.

[13] De Greef J., Belpaeme T. (2015). Why Robots Should Be Social: Enhancing Machine Learning through Social Human-Robot Interaction. PLoS ONE.

[14] Kennedy J., Baxter P., Belpaeme T. (2017). The Impact of Robot Tutor Nonverbal Social Behavior on Child Learning. Front. Ict.

[15] Serholt S. Child–Robot Interaction in Education. Gothenburg: University of Gothenburg 2017. http://hdl.handle.net/2077/52564.

[16] Takanishi A., Endo N., Petersen K. (2012). Towards Natural Emotional Expression and Interaction: Development of Anthropomorphic Emotion Expression and Interaction Robots. Int. J. Synth. Emot (IJSE).

[17] Dieh J., Schmitt L., Villano M., Crowell C. (2012). The clinical use of robots for individuals with Autism Spectrum Disoirders: A critical Review. Res. Autism C2A0Spectr. Disord..

[18] Scassellati B., Admoni H., Mataric M. (2012). Robots for Use in Autism Research. Annu. Rev. Biomed. Eng..

[19] Cao H.-L., Esteban P.G., Bartlett M., Baxter P., Belpaeme T., Billing E., Cai H., Coeckelbergh M., Costescu C., David D. (2019). Robot-EnhancedTherapy. Development and Validation Of Supervised Autonomous Robotic System For Autism Spectrum Disorders Therapy. IEEE Robot. Autom. Mag..

[20] García-Varea I., Jiménez-Picazo A., Martínez-Gómez J., Revuelta-Martínez A., Rodríguez-Ruiz L., Bustos P., Nuñez-Trujillo P.M. APEDROS: Asistencia a Personas con Discapacidad mediante Robots Sociales. Retrieved from VI Congreso Iberoamericano de Tecnologias de Apoyo a la Discapacidad IBERDISCAP June 2011. https://www.researchgate.net/publication/235938148_APEDROS_ASISTENCIA_A_PERSONAS_CON_DISCAPACIDAD_MEDIANTE_ROBOTS_SOCIALES.

[21] Lee H., Hyun E. (2015). The Intelligent Robot Contents for Children with Speech-Language Disorder. J. Educ. Techno. Soc..

[22] Peñeñory V.M., Manresa-Yee C., Riquelme I., Collazos C.A., Fardoun H.M. (2018). Scoping Review of Systems to Train Psychomotor Skills in Hearing Impaired Children. Sensors.

[23] Leyzberg D., Spaulding S., Scassellati B. Personalizing robot tutors to individuals′ learning differences. Proceedings of the 2014 ACM/IEEE International Conference on Human-Robot Interaction.

[24] Westlund J.M., Dickens L., Jeong S., Harris P.L., DeSteno D., Breazeala C.L. (2017). Children Use Non-Verbal Cues to Learn New Words From Robots as well as People. Int. J. Child. Comput. Interact..

[25] Cherney L.R., Halper A.S., Holland A.L., Cole R. (2008). Computerized script training for aphasia: Preliminary results. Am. J. Speech Lang. Pathol..

[26] Jeon K.H., Yeon S.J., Kim Y.T., Song S., Kim J. Robot-based augmentative and alternative communication for nonverbal children with communication disorders. Proceedings of the 2014 ACM International Joint Conference on Pervasive and Ubiquitous Computing (UbiComp ′14).

[27] Malik N.A., Yussof H., Hanapiah F.A. (2014). Development of Imitation Learning through Physical Therapy Using a Humanoid Robot. Procedia Comput. Sci..

[28] Nehaniv C.L., Dautenhahn K. (2007). Imitation and Social Learning in ROBOTS, HUMANS and Animals: Behavioural, Social and Communicative Dimensions.

[29] Dautenhahn K., Werry I. A Quantitative technique for analysing robot-human. Proceedings of the IROS2002, IEEE/RSJ International Conference on Intelligent Robots.

[30] Kennedy J., Baxter P., Belpaeme T. (2017). Nonverbal Immediacy as a Characterisation of Social Behaviour for Human–Robot Interaction. Int. J. Soc. Robot..

[31] Serholt S. (2017). Breakdowns in children’s interactions with a robotic tutor: A longitudinal study. Comput. Hum. Behav..

[32] Beukelman D.R., Light J.C. (2020). Augmentative and Alternative Communication: Supporting Children and Adults with Complex Communication Needs.

[33] Vasalou A., Khaled R., Holmes W., Gooch D. (2017). Digital games-based learning for children with dyslexia: A social constructivist perspective on engagement and learning during group game-play. Comput. Educ..

[34] Gouaillier D., Hugel V., Blazevic P., Kilner C., Monceaux J., Lafourcade P., Marnier B., Serre J., Maisonnier B. (2008). The NAO humanoid: A combination of performance and affordability. CoRR.

[35] Gomilko S., Zimina A., Shandarov E., Ronzhin A., Rigoll G., Meshcheryakov R. (2016). Attention Training Game with Aldebaran Robotics NAO and Brain-Computer Interface. Interactive Collaborative Robotics.

[36] Nalin M., Bergamini L., Giusti A., Baroni I., Sanna A. Children’s perception of a Robotic Companion in a mildly constrained setting. Proceedings of IEEE/ACM Human-Robot Interaction 2011 Conference (Robots with Children Workshop).

[37] Ismail L., Shamsudin S., Yussof H., Hanapiah F., Zahari N.I. (2012). Robot-based Intervention Program for Autistic Children with Humanoid Robot NAO: Initial Response in Stereotyped Behavior. Procedia Eng..

[38] Vrochidou E., Najoua A., Lytridis C., Salonidis M., Ferelis V., Papakostas G. Social robot NAO as a self-regulating didactic mediator: A case study of teaching/learning numeracy. Proceedings of the 26th International Conference on Software, Telecommunications and Computer Networks (SoftCOM).

[39] Andreasson R., Alenljung B., Billing E., Lowe R. (2018). Affective Touch in Human–Robot Interaction: Conveying Emotion to the Nao Robot. Int. J. Soc. Robot..

[40] Alenljung B., Andreasson R., Lowe R., Billing E., Lindblom J. (2018). Conveying emotions by touch to the Nao Robot: A user experience perspective. Multimod. Technol. Interact..

[41] Rossi S., Larafa M., Ruocco M. (2019). Emotional and Behavioural Distraction by a Social Robot for Children Anxiety Reduction During Vaccination. Int. J. Soc. Robot..

[42] Dahl T., Kamel Boulos M.N. (2013). Robots in health and social care: A complementary technology to home care and telehealthcare?. Robotics.

[43] Huisman C., Kort H. (2019). Two-year use of care robot Zora in Dutch nursing homes: An evaluation study. Healthcare.

[44] Taheri A., Meghdari A., Alemi M., Pouretemad H. (2018). Human–Robot Interaction in Autism Treatment: A Case Study on Three Pairs of Autistic Children as Twins, Siblings, and Classmates. Int. J. Soc. Robot..

[45] Tapus A., Peca A., Aly A., Pop C., Jisa L., Pintea S., Rusu A., David D. (2012). Children with autism social engagement in interaction with Nao, an imitative robot—A series of single case experiments. Interact. Stud..

[46] Yin R.K. (2003). Case Study Research: Design and Methods.

[47] Stake R.E. (1995). The Art of Case Study Research.

